# Comparative Analysis of 3D Cephalometry Provided with Artificial Intelligence and Manual Tracing

**DOI:** 10.3390/diagnostics14222524

**Published:** 2024-11-12

**Authors:** Zurab Khabadze, Oleg Mordanov, Ekaterina Shilyaeva

**Affiliations:** Department of Therapeutic Dentistry, Peoples’ Friendship University of Russia Named after Patrice Lumumba (RUDN University), 6 Miklukho-Maklaya St., 117198 Moscow, Russia; khabadze-zs@rudn.ru (Z.K.); shilyaeva-es@rudn.ru (E.S.)

**Keywords:** artificial intelligence, 3D cephalometry, Diagnocat

## Abstract

Objectives: To compare 3D cephalometric analysis performed using AI with that conducted manually by a specialist orthodontist. Methods: The CBCT scans (a field of view of 15 × 15 cm) used in the study were obtained from 30 consecutive patients, aged 18 to 50. The 3D cephalometric analysis was conducted using two methods. The first method involved manual tracing performed with the Invivo 6 software (Anatomage Inc., Santa Clara, CA, USA). The second method involved using AI for cephalometric measurements as part of an orthodontic report generated by the Diagnocat system (Diagnocat Ltd., San Francisco, CA, USA). Results: A statistically significant difference within one standard deviation of the parameter was found in the following measurements: SNA, SNB, and the left interincisal angle. Statistically significant differences within two standard deviations were noted in the following measurements: the right and left gonial angles, the left upper incisor, and the right lower incisor. No statistically significant differences were observed beyond two standard deviations. Conclusions: AI in the form of Diagnocat proved to be effective in assessing the mandibular growth direction, defining the skeletal class, and estimating the overbite, overjet, and Wits parameter.

## 1. Introduction

Since its inception, artificial intelligence (AI) has led to significant advancements, enhancing various aspects of our daily lives, including medical practices [1,2]. Recently, AI has facilitated the development of personalized medicine, enabling more precise disease predisposition assessments and diagnoses and the selection of optimal treatments for individuals [3]. In dentistry, AI is employed for various tasks, including numbering teeth, analyzing the relationship between the third molars and the mandibular canal, planning dental implants, diagnosing periapical pathologies, detecting cavities, evaluating osteoporotic changes, and examining jaw tumors [4].

AI is frequently employed in the processing and analysis of cone beam computed tomography (CBCT) in dentistry [5]. Advanced AI algorithms, like Diagnocat, have been created to facilitate both the coarse and fine volume segmentation of teeth in CBCT images, making them highly effective in managing large datasets [6]. Diagnocat’s AI efficiently analyzes CBCT images in DICOM format, ensuring smooth data transfer [7]. AI-powered dental imaging software significantly enhances the speed and efficiency of data processing [8]. CBCT, a 3D imaging technology, is widely used in dentistry for various applications, including implant placement, orthodontic treatment planning, and root canal procedures [9]. Currently, considerable scientific effort is being directed towards AI applications that aim to automate the identification of landmarks in CBCT [10].

Cephalometric analysis is a quantitative diagnostic tool routinely utilized by orthodontists, prosthodontists, and maxillofacial and orthognatic surgeons to assess skeletal and dentoalveolar relationships, morphometric characteristics, and growth patterns in patients. Since its introduction in 1931, this method has evolved significantly, integrating the latest advancements in orthodontic radiology and diagnostics [11]. The analysis relies on linear and angular measurements derived from two-dimensional (2D) radiographs of the skull, producing a personalized cephalogram for each patient [12]. Traditional cephalometric reference points are identified on skeletal structures, such as the anterior and posterior cranial base, maxilla, and mandible; on teeth, including the molars and incisors; and on soft tissue structures, such as the nose and chin. By measuring the distances and angles between these landmarks and axes, clinicians can categorize patients according to their skeletal, dental, and profilometric features. Despite technological advancements, the manual tracing of specific points in relation to key anatomical structures of the skull and neck on lateral, frontal, and axial 2D radiographs remains the gold standard for this procedure. The primary challenges in accurately identifying cephalometric points include the time commitment, the high level of expertise required, and the potential for variability between and within operators [13].

While software is now widely utilized for cephalometric measurements, the tracing of landmarks continues to be a manual process that must be carried out by an orthodontic specialist [14]. The accuracy of this analysis heavily depends on the expert’s experience and even their condition on a given day, which can lead to inconsistencies [15]. The lack of reliability in manual tracing is a significant concern, as the inaccurate identification of cephalometric landmarks can result in incorrect decisions in orthodontic treatment. Therefore, the development of fully automated and reliable methods for the identification of these landmarks is highly sought after to enhance quality control. AI algorithms offer promising new opportunities to assist orthodontic professionals in their daily work, potentially improving both efficiency and accuracy [16].

To the authors’ knowledge, there have been only a limited number of studies that have employed convolutional neural networks (CNNs) for automated 3D cephalometric analysis. Although these initial studies have shown promising results, they have also revealed significant methodological limitations [17]. Consequently, definitive conclusions about the practical applicability of these algorithms are still not well established.

The aim of this study is to compare 3D cephalometric analysis performed using AI with that conducted manually by a specialist orthodontist and maxillofacial radiology specialist. The null hypothesis is the following: there is no significant difference between 3D cephalometric analysis performed using an AI-powered virtual assistant and that conducted manually by a human orthodontist.

## 2. Materials and Methods

This study received approval from the bioethical committee of RUDN University (Protocol No. 12, 17 March 2024). It was based on a retrospective and registration dataset, meaning that it did not involve human experiments or the use of human tissue samples, and no patients were specifically imaged for this research.

### 2.1. Study Design and Patient Selection

The CBCT scans used in this study were obtained from 30 consecutive patients, aged 18 to 50 (13 males and 17 females), who were admitted to a local diagnostic center. The sample was annotated by a dental and maxillofacial radiology specialist with extensive experience in surgical procedures and full-mouth rehabilitation and an orthodontist using cephalometric landmarks. Both specialists provided the tracing twice (7 days between measurements) to calibrate the results. Samples were excluded if the difference in the measurements between the specialists was more than half of a standard deviation or more than half of a standard deviation within the measurements of one specialist. These procedures were provided for expert calibration. As a result, none of the patients were excluded and the diagnostic outcome for each parameter was similar. Then, the mean value of the measurements was calculated. The general inclusion criteria for the study were as follows: patients with well-controlled systemic diseases, patients requiring cephalometric radiographs and CBCT, patients without maxillofacial deformities, patients with fully erupted incisors and first molars, and patients without any previous orthodontic treatment.

All CBCT images were obtained using the GALILEOS Comfort system (Sirona Dental Systems GmbH, Bensheim, Germany) with the following acquisition settings: tube voltage of 98 kV, tube current of 5 mAs, scanning time of 14 s, field of view (FOV) of 15 × 15 cm, and isotropic voxel size of 0.25 mm. During the scan, the patients were positioned in habitual occlusion, with their lips and tongue at rest. Their heads were stabilized using head and chin supports, ensuring that no excessive pressure was applied.

### 2.2. 3D Cephalometric Analysis

**Figure 1:**
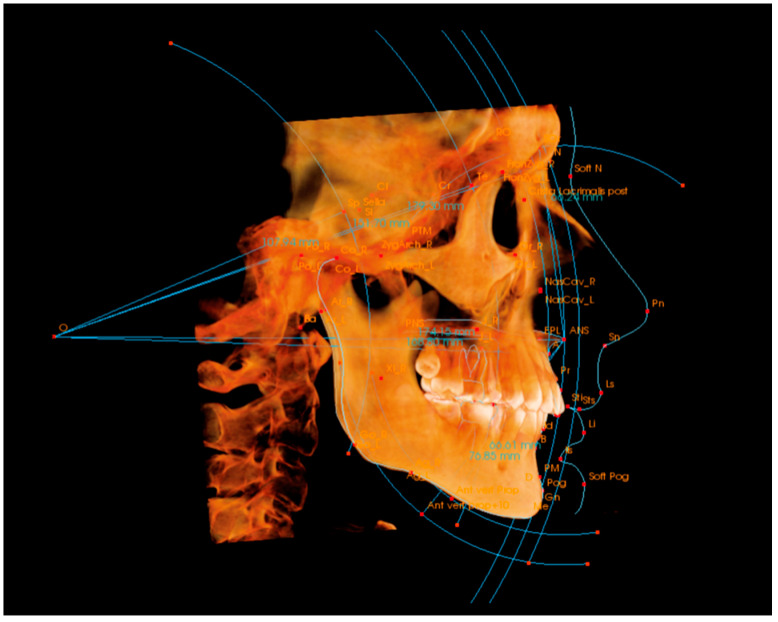
Manual 3D cephalometry tracings.

The 3D cephalometric analysis was conducted using two methods. The first method involved manual tracing performed with the Invivo 6 software (Anatomage Inc., Santa Clara, CA, USA). The number of landmarks was predetermined to ensure consistency in the cephalometric measurements across the methods studied. Initially, the CBCT images were aligned using specific landmarks: Ba, Or R, Or L, Po R, and Na. Following this alignment, all landmarks were traced according to the established definitions [18,19] (Figure 1).

**Figure 2:**
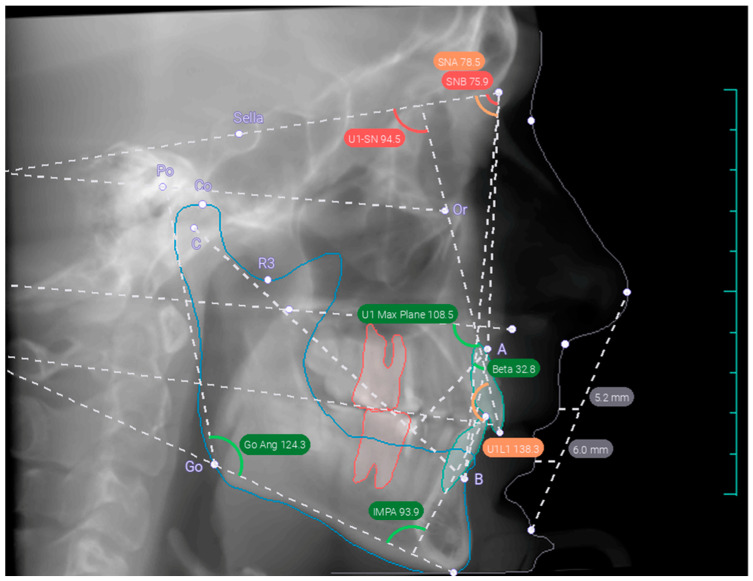
The 3D cephalometry tracings obtained with AI Diagnocat.

The second method involved using AI for cephalometric measurements as part of an orthodontic report generated by the Diagnocat system (Diagnocat Ltd., San Francisco, CA, USA). The Diagnocat AI system produces an orthodontic report by utilizing a pipeline composed of multiple pre-trained fully convolutional networks, along with algorithmic slice extraction and AI-driven tracings (Figure 2). This system leverages a set of pre-trained semantic segmentation networks, which are based on an internally modified fully convolutional 3D U-Net architecture, to achieve voxel-perfect segmentation masks of the teeth and anatomical elements present in the images [20].

The cephalometric measurements are detailed in Table 1. Each result was evaluated separately and independently, without any prior knowledge of the AI results, to determine the reliability of the AI-generated diagnostic reports.

### 2.3. Statistical Analysis

The R language for statistical computing was used for the data analysis. Illustrations were created using the package “ggplot2”. Quantitative variables were represented with the minimum (min), maximum (max), median (M), first (Q1) and third (Q3) quartiles, average/mean (mean), and standard deviation (SD). Statistical comparisons were carried out using Student’s *t*-test for normally distributed quantitative variables and the Mann–Whitney U test for non-normally distributed quantitative variables.

Categorical data were represented with fractions and percentages. For categorical data, statistical comparisons were carried out using Fisher’s exact test. The agreement between the methods was estimated using the intraclass correlation coefficient (ICC). This utilized a two-factor analysis of variance as implemented in the package irr. The statistical significance cutoff was selected as *p* < 0.05. No correction for multiple comparisons was carried out.

## 3. Results

**Figure 3:**
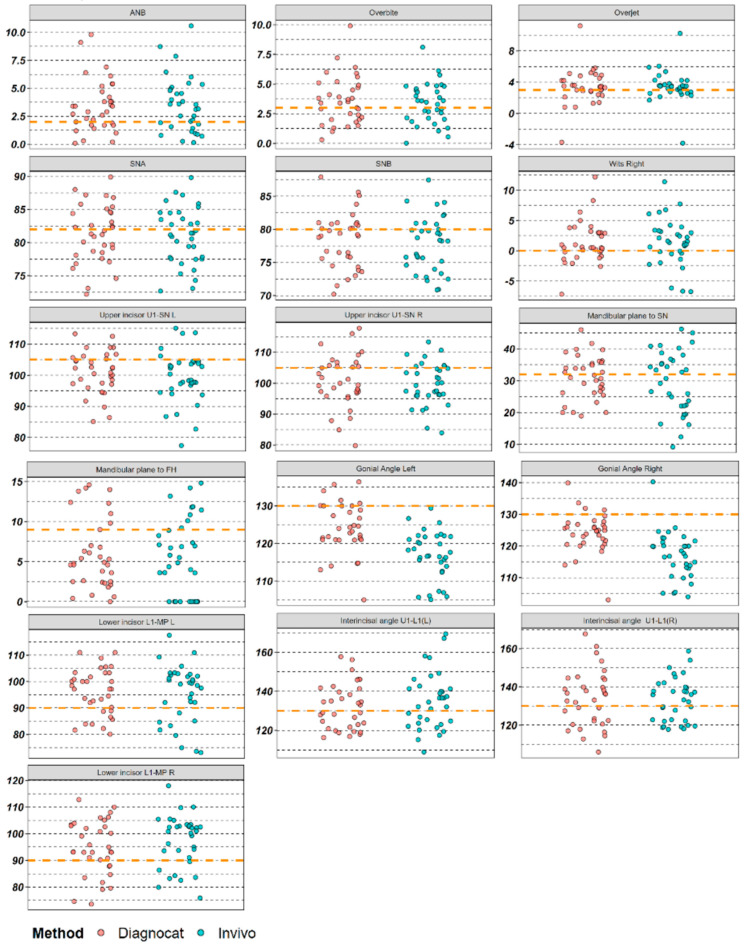
Overview plot. Orange dotted line is the expected value for the given parameter. Units of measurement are individual per variable.

**Figure 4:**
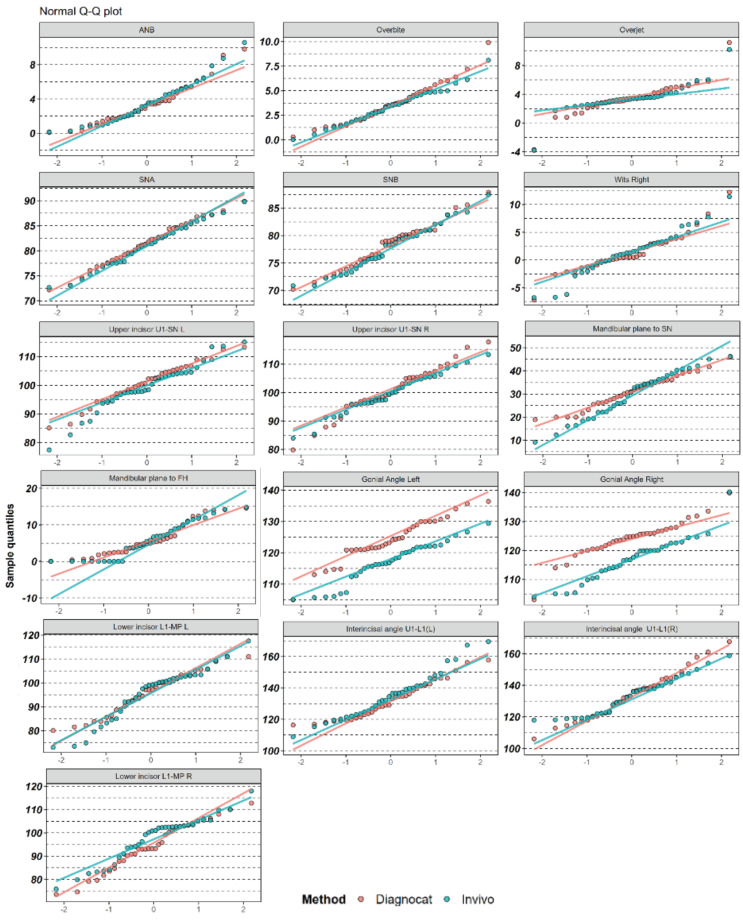
Q-Q plots for each variable (by method). The better the alignment of the dots with the line of the corresponding color, the closer the distribution to normality.

**Figure 5:**
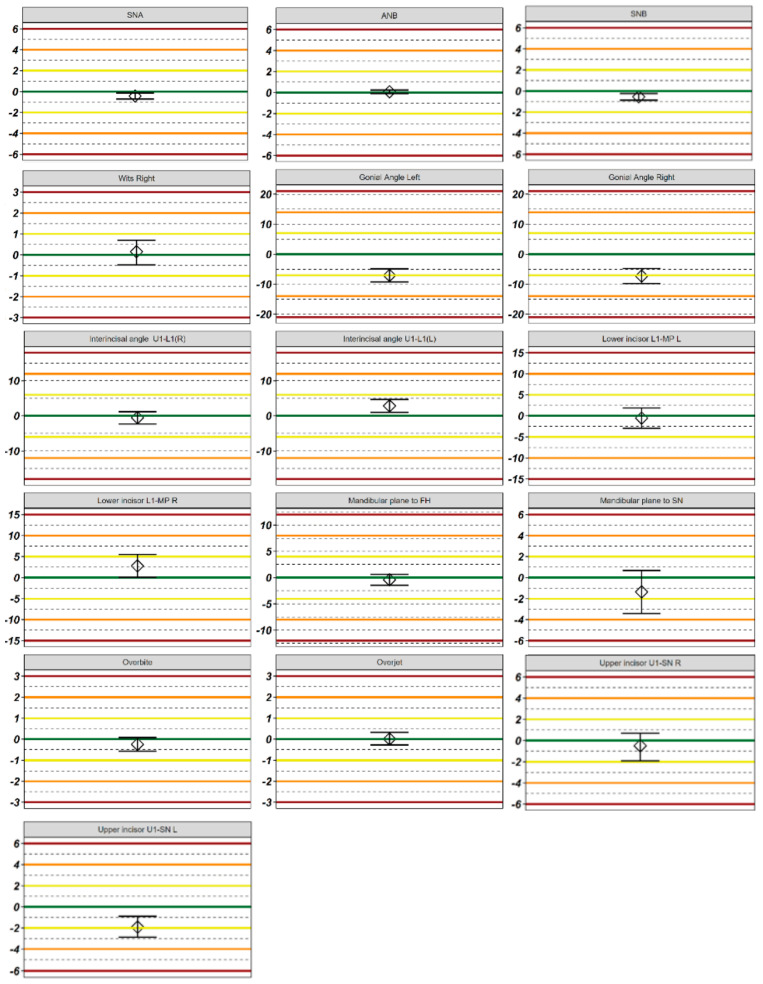
Differences between the methods of Diagnocat and Invivo for each variable. In the case of a normal distribution, parametric methods were used, Student’s *t*-test was used for comparisons, the central diamond represents the average/mean value of the difference, and the whiskers denote the parametric 95% confidence interval for it. In the case of a non-normal distribution, non-parametric methods were used, the Mann–Whitney U test was used for comparisons, the central diamond represents the pseudomedian of the difference, and the whiskers denote the non-parametric 95% confidence interval for it. Green line—zero; yellow lines—one standard deviation of the method; orange lines—two standard deviations of the method; red lines—three standard deviations of the method. Units of measurement are individual per variable.

In the initial stage of the study, the data were organized into a visual format to identify any CBCT scans where the two methods did not correlate. This approach, described in the Materials and Methods section, was used to ensure that 3D cephalometry was accurately performed by both methods (Figure 3). In the subsequent stage, Q-Q plots were generated to validate the statistical parameters (Figure 4). Table 2 and Figure 5 illustrate the differences between the Diagnocat and Invivo methods for each variable. Depending on the results of the Shapiro–Wilk test and the examination of the normal Q-Q plots, either parametric or non-parametric methods were applied.

When comparing the measured values between manual tracing and AI, statistically significant differences were observed. A statistically significant difference within one standard deviation of the parameter was found in the following measurements: SNA, SNB, and the left interincisal angle. Statistically significant differences within two standard deviations were noted in the following measurements: the right and left gonial angles, the left upper incisor, and the right lower incisor. No statistically significant differences were observed beyond two standard deviations.

## 4. Discussion

CBCT is increasingly utilized in orthodontics because of its capability to provide detailed three-dimensional (3D) images of dental structures, soft tissue, nerve pathways, and bone [20]. This study compared the automated 3D cephalometric analysis performed using AI Diagnocat with manual tracings performed using Invivo 6. Recent studies have consistently demonstrated the superior accuracy of 3D cephalometric analysis over traditional 2D methods [21], as well as the enhanced efficiency of deep learning (DL) algorithms compared to conventional machine learning techniques in the field of bioimaging [22]. Consequently, there is a growing trend toward developing DL-based algorithms for the automatic identification of landmarks in 3D images.

Several reviews and meta-analyses have explored the use of AI in 3D cephalometric analysis within orthodontics [17,23]. Deep learning (DL) algorithms have consistently demonstrated greater accuracy in automated 3D cephalometric landmark identification compared to other machine learning (ML) algorithms. Over the years, promising DL models have been developed, leading to significant improvements in the accuracy of landmark annotation. However, the main limitations of the existing reviews and meta-analyses stem from the studies included for qualitative and quantitative assessment, which do not feature Diagnocat. Additionally, the practical application of such AI algorithms in routine orthodontic practice remains underexplored, as previous studies have primarily focused on landmark detection, without adequately assessing the accuracy of the cephalometric parameters [24] or providing sufficient statistical evaluation.

This study identified statistically significant differences in the following parameters: SNA, SNB, left interincisal angle, right and left gonial angles, left upper incisor, and right lower incisor. Cephalometric analysis typically represents a three-dimensional (3D) structure in a two-dimensional (2D) format. As a result, these measurements on radiographic images can be prone to projection and measurement errors, as well as individual variations [25]. Thus, the null hypothesis is rejected.

The SNA and SNB are angles that measure the relationship between the SN plane and the NA or NB points, respectively. These angles have long been recognized as important indicators of upper and lower facial prognathism and are valuable tools in diagnosing and treating malocclusion [26]. Ariwa et al. [27] observed differences between conventional cephalometric analysis and 3D analysis using CBCT in measuring the ANB angle, which reflects the anteroposterior intermaxillary relationship by connecting points A, N, and B. The regression equations from their study highlighted the influence of the A-point plot. However, our study found no significant difference in the ANB angle (*p* = 0.347), indicating that both methods provided consistent definitions of the skeletal class.

The differences observed in the SNA and SNB angles in our study might be attributed to variations in defining the S-point (midpoint of the shadow of the sella turcica). Additionally, in 3D analysis, unlike traditional cephalometrics, the ANB angle may not simply represent the difference between the SNA and SNB [28]. When 3D cephalometric measurements are projected onto the midsagittal plane, they retain the same significance as in 2D cephalometry. However, traditional cephalometric indices may not fully capture the nuances of 3D analysis using CBCT [27]. Das et al. [25] concluded that cephalometric landmarks that are challenging to identify on 2D cephalograms can be more accurately and reliably located and measured on 3D CBCT-generated cephalograms. Both angular and linear measurements tend to be significantly greater in 3D CBCT-generated values

Regarding the incisor parameters, the statistically significant difference was unilateral. Kunz et al. [29] compared the accuracy of AI-based analysis to the current gold standard—analyses performed by human experts—to assess its precision and clinical applicability in routine orthodontic practice. They found that the inclination of the upper incisors showed a statistically higher absolute difference of 2.18° between the AI’s predictions and the human experts’ gold standard, compared to a mean absolute difference of 1.50° between the human examiners and the gold standard. The accurate placement of anatomical cephalometric landmarks is crucial for the linear, angular, and planar comparison of the selected landmarks, which is essential in planning both orthodontic and orthognathic surgery procedures.

Chen et al. [30] studied the skill involved in placing anatomical and cephalometric points and concluded that specialists with more years of active clinical practice demonstrated greater proficiency in accurately placing these reference points for precise cephalometric analysis [31]. It is also important to consider that clinicians, dentists, surgeons, and orthodontists who regularly perform these analyses have more experience compared to those who do so only sporadically. Despite a well-conducted analysis, errors can still occur. For example, Zamrik et al. [32] highlighted such an instance in their study, where the measurement of the U1-A point was inaccurately performed.

In terms of the effectiveness of individual programs, some studies did not find significant differences between analyses conducted by software and those performed by a specialist [33]. Kunz et al. [29] noted that it could be seen as an “unfair” competition for the AI, given that the human raters themselves establish the gold standard used for comparison.

The statistically significant difference observed in the bilateral gonial angle may be attributed to the standardization of landmarks. The gonion is a specific point on the contour of the mandible, identified by bisecting the angle formed by the mandibular and ramus planes [34]. The overall localization error reported in various studies reflects the different types and numbers of annotated landmarks, ranging from 5 to 105 [17]. Currently, there is no standardized threshold for localization errors in 3D cephalometric analysis, and the required accuracy can vary depending on the positioning and type of the landmark, whether anatomical or geometrical.

The quality of landmark identification and their precise placement is critical to the reliability of 3D linear and angular measurements [35]. For a landmark to be used effectively in evaluating a particular dimension, it must demonstrate good consistency and precision. Although manual identification is considered the gold standard, it is susceptible to human error, which is not always quantified in the literature. Nonetheless, from a clinical perspective, the repeatability and reproducibility of manually placing landmarks in 3D images are generally acceptable for most anatomical reference points [17].

Moreover, because 2D cephalometry remains the gold standard, there is no universally defined set of points for 3D analysis [36]. The studies reviewed often used different landmarks, making it difficult to precisely compare the performance of landmark annotations across various algorithms. Alsubai [24] concluded that existing research on AI-based cephalometric landmark annotation, reliability, and accuracy in automatic 3D cephalometric landmarking has generally assessed these factors at an equal rate of 6%.

AI-driven tools have the potential to streamline workflows in dental practices by automating tasks such as image registration, data management, and report generation [37]. This automation enables dental professionals to dedicate more time to patient care and less to administrative duties. Various studies have utilized the Diagnocat software to investigate different aspects of dental practice [38,39,40]. For instance, one study successfully explored the software’s ability to detect periapical lesions. Another study by Orhan et al. [39] examined the effectiveness of AI in diagnosing impacted third molars, assessing their relationship with adjacent anatomical structures and determining the number of root canals.

While Diagnocat offers a range of reports that have been studied in various contexts, there has been no specific research focused on its use in generating orthodontic reports. Although the minimal field of view (FOV) for Diagnocat’s CBCT analysis is 15 × 13 cm, it is still capable of producing the necessary parameters within the reduced volume of the CBCT scan. Kissel et al. [41] demonstrated that minimized large FOVs could meet the requirements for 3D cephalometric analyses using the caudal reference plane, such as the FH plane. In these cases, Diagnocat proves to be a useful tool as well.

The first limitation encountered during the study was the insufficient number of specialists involved. The experience and expertise of clinicians, as well as the proficiency of younger clinicians, significantly influence the accuracy of cephalometric tracings [42]. Manual tracings depend heavily on the clinician’s experience and knowledge to accurately identify craniometric reference points, whereas automatic or AI-driven software processes images based on algorithms and other automated tools. Specialists with more years of active clinical practice tend to be more proficient in accurately placing the necessary reference points for precise cephalometric analysis [31]. Moreover, clinicians, dentists, surgeons, and orthodontists who perform such analyses regularly have more experience than those who do so only occasionally.

Another issue identified in some studies is the reproducibility crisis in AI, where the same research results cannot be consistently replicated if the experiment is performed by a different set of researchers. This issue stems from deficiencies in metric knowledge and algorithm design. Additionally, many researchers overlook the sensitivity of the results to various hyper-parameters, including the initialization strategy, iteration times, and learning rates [43,44].

The second limitation is the insufficiency of Diagnocat’s data. Inconsistencies in the training data raise questions about the reliability of various algorithmic models. While supervised models are preferred for the diagnosis of malocclusions, the high costs and the need for extensive labeling make it challenging to create high-quality, standardized datasets for orthodontics [44].

The third limitation is the reduced number of parameters available in Diagnocat. This limitation restricts the ability to separate several parameters into distinct groups, such as the growth direction, skeletal class, and others.

## 5. Conclusions

This study compared 3D cephalometric analyses conducted by specialists with those performed using artificial intelligence. The findings indicated an acceptable correlation between the two methods. The AI tool Diagnocat proved to be effective in assessing the mandibular growth direction, defining the skeletal class, and estimating the overbite, overjet, and Wits parameter. However, discrepancies in other parameters, such as those describing the upper and lower jaw positions, the incisor inclination, and the angle of the mandibula, could potentially be addressed by standardizing the guidelines for both AI and manual tracings. Additionally, since the exact methodology of the AI tool Diagnocat for 3D cephalometry is not fully understood, it remains unclear whether the measurement inaccuracies stem from the AI, the specialist, or both.

## Figures and Tables

**Table 1:** The measurements and norms of the compared parameters (values are taken from the current Diagnocat orthoreport).

Variable	Norm	SD
Mandibular plane to SN	32°	2
Mandibular plane to FH	9°	4
Gonial angle right	130°	7
Gonial angle left	130°	7
Wits	0 mm	1
SNA	82°	2
SNB	80°	2
ANB	2°	2
Overjet	3 mm	1
Overbite	3 mm	1
Upper incisor U1-SN R	105°	2
Upper incisor U1-SN L	105°	2
Lower incisor L1-MP R	90°	5
Lower incisor L1-MP L	90°	5
Interincisal angle U1-L1(R)	130°	6
Interincisal angle U1-L1(L)	130°	6

**Table 2:** Differences between the methods of Diagnocat and Invivo for each variable. LCL—lower limit of the 95% confidence interval for the difference. UCL—upper limit of the 95% confidence interval for the difference. SD—standard deviation. In the case of a normal distribution, parametric methods were used, Student’s *t*-test was used for comparisons, the difference was represented by the average/mean value, and the 95% confidence interval for it was parametric. In the case of a non-normal distribution, non-parametric methods were used, the Mann–Whitney U test was used for comparisons, the difference was represented by the pseudomedian, and the 95% confidence interval for it was non-parametric. Values of *p* less than 0.05 * indicate a statistically significant difference between the methods of Diagnocat and Invivo.

Variable	Method	Difference	LCL	UCL	*p*
Mandibular plane to SN	parametric	−1.36	−3.42	0.69	0.186
Mandibular plane to FH	non-parametric	−0.42	−1.47	0.60	0.411
Gonial angle right	parametric	−7.29	−9.82	−4.77	<0.001 *
Gonial angle left	parametric	−7.07	−9.27	−4.87	<0.001 *
Wits right	non-parametric	0.15	−0.48	0.70	0.648
SNA	parametric	−0.43	−0.72	−0.13	0.006 *
SNB	parametric	−0.55	−0.87	−0.23	0.001 *
ANB	non-parametric	0.08	−0.09	0.25	0.347
Overjet	non-parametric	0.01	−0.27	0.34	0.945
Overbite	parametric	−0.25	−0.57	0.07	0.126
Upper incisor U1-SN R	non-parametric	−0.49	−1.91	0.69	0.447
Upper incisor U1-SN L	non-parametric	−1.92	−2.88	−0.90	0.001 *
Lower incisor L1-MP R	parametric	2.77	0.03	5.51	0.048 *
Lower incisor L1-MP L	parametric	−0.56	−2.96	1.84	0.637
Interincisal angle U1-L1(R)	parametric	−0.56	−2.30	1.19	0.519
Interincisal angle U1-L1(L)	parametric	2.82	0.97	4.67	0.004

## Data Availability

All data are available on reasonable request from the corresponding author.
